# Radiolabeled 15-mer peptide internalization is mediated by megalin (LRP2 receptor) in a CRISPR/Cas9-based *LRP2* knockout human kidney cell model

**DOI:** 10.1186/s41181-024-00262-2

**Published:** 2024-04-18

**Authors:** Anna Durinova, Lucie Smutna, Pavel Barta, Rajamanikkam Kamaraj, Tomas Smutny, Bernhard Schmierer, Petr Pavek, Frantisek Trejtnar

**Affiliations:** 1 Division of Radiopharmacy, Department of Pharmacology and Toxicology, Faculty of Pharmacy in Hradec Kralove, Charles University, Hradec Kralove, Czech Republic; 2 Department of Biophysics and Physical Chemistry, Faculty of Pharmacy in Hradec Kralove, Charles University, Hradec Kralove, Czech Republic; 3 SciLifeLab and Department of Medical Biochemistry and Biophysics, CRISPR Functional Genomics, Karolinska Institutet, Solna, Sweden

**Keywords:** Radiolabeled peptide, LRP2, Megalin, CRISPR/Cas9, Radionephrotoxicity

## Abstract

**Background:**

Megalin (LRP2 receptor) mediates the endocytosis of radiolabeled peptides into proximal tubular kidney cells, which may cause nephrotoxicity due to the accumulation of a radioactive tracer. The study aimed to develop a cellular model of human kidney HK2 cells with *LRP2* knockout (KO) using CRISPR/Cas9 technique. This model was employed for the determination of the megalin-mediated accumulation of ^68^Ga- and ^99m^Tc-labeled 15-mer peptide developed to target the vascular endothelial growth factor (VEGF) receptor in oncology radiodiagnostics.

**Results:**

The gene editing in the *LRP2* KO model was verified by testing two well-known megalin ligands when higher viability of KO cells was observed after gentamicin treatment at cytotoxic concentrations and lower FITC-albumin internalization by the KO cells was detected in accumulation studies. Fluorescent-activated cell sorting was used to separate genetically modified *LRP2* KO cell subpopulations. Moreover, flow cytometry with a specific antibody against megalin confirmed *LRP2* knockout. The verified KO model identified both ^68^Ga- and ^99m^Tc-radiolabeled 15-mer peptides as megalin ligands in accumulation studies. We found that both radiolabeled 15-mers enter *LRP2* KO HK2 cells to a lesser extent compared to parent cells. Differences in megalin-mediated cellular uptake depending on the radiolabeling were not observed. Using biomolecular docking, the interaction site of the 15-mer with megalin was also described.

**Conclusion:**

The CRISPR/Cas9 knockout of *LRP2* in human kidney HK2 cells is an effective approach for the determination of radiopeptide internalization mediated by megalin. This in vitro method provided direct molecular evidence for the cellular uptake of radiolabeled anti-VEGFR 15-mer peptides via megalin.

**Graphical abstract:**

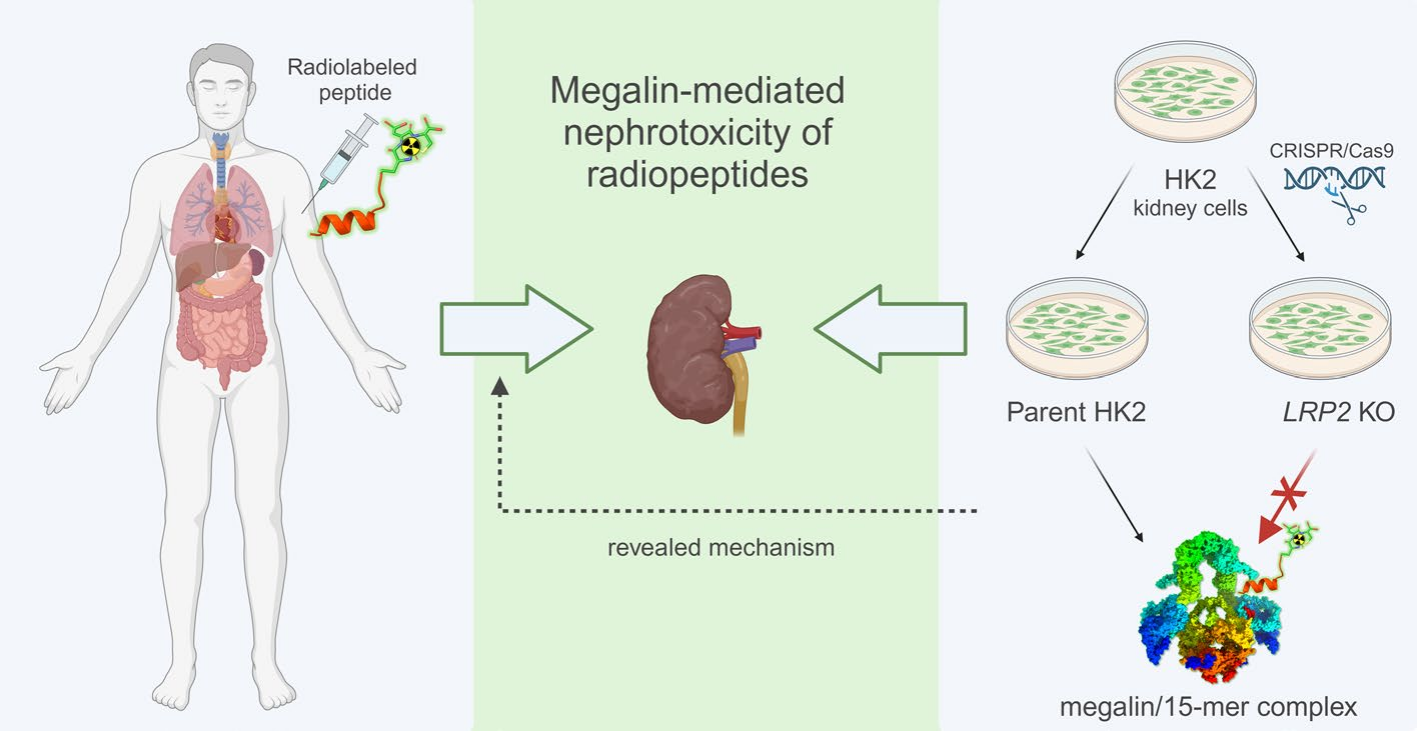

**Supplementary Information:**

The online version contains supplementary material available at 10.1186/s41181-024-00262-2.

## Background

Radiolabeled receptor-specific peptides represent a state-of-art nuclear medicine tool applied in both the diagnosis and treatment of oncologic and other serious diseases (Kręcisz et al. 2021). One of the key pharmacokinetic characteristics of radiolabeled peptides is their rapid renal excretion. The effective removal from the systemic circulation limits the background radioactivity of radiodiagnostic peptides and protects non-target tissues from undesired radiation exposure or even radiotoxic damage (Geenen et al. 2021). Nevertheless, radiopeptides filtrated into urine can be subsequently reabsorbed from the ultrafiltrate into proximal tubular cells, where they are transferred into lysosomes and degraded by proteolytic enzymes. Tubular accumulation and extended retention of the radiopeptide fragments may result in consequent radiotoxic renal damage (Akizawa et al. 2008). Hence, the development of novel receptor-specific radiopeptide agents should include the selection of candidates with minimal internalization into the renal tubular cells. Therefore, the identification of mechanisms involved in kidney retention is an important step in radiopeptide preclinical testing. Moreover, based on the 3Rs (replacement, reduction, refinement) principle in animal testing, appropriate in vitro methods are desirable to be introduced for effective screening and selection among the series of radiopeptide candidates.

Several mechanisms may potentially contribute to radiopeptide transport across the cell membrane (Volková et al. 2015). Previous experimental studies have suggested that megalin (LRP2, low-density lipoprotein receptor-related protein 2) may be one of the most important transport mechanisms involved in radiopeptide renal accumulation (Vegt et al. 2011). Megalin, the endocytic receptor, is localized in a wide variety of tissues, the megalin-mediated endocytosis is especially important in the kidney, where it is responsible for the reabsorption of various compounds from urine, including proteins such as albumin or drugs such as aminoglycoside antibiotics (Marzolo and Farfán 2011; Goto et al. 2023). The megalin cooperates with another membrane protein the cubilin (*CUBN*) coreceptor. In contrast to megalin, cubilin lacks a transmembrane domain and thus is not responsible for endocytosis by itself. Forming complex of cubilin, megalin, and appropriate ligand leads to internalization (Roode et al. 2024).

The study of megalin interaction with radiopeptides has remained limited due to the absence of a relevant in vitro model. The development of a human kidney model which would aid in the identification of megalin radioligands represents an unsolved research challenge towards the development of radiolabeled receptor-specific peptides for molecular imaging and/or targeted radiotherapy.

In the project, we focused on the development of a reliable cellular model using the CRISPR/Cas9 technique to knockout the *LRP2* gene by editing the crucial motifs for megalin function in human kidney HK2 cells. The knockout function was verified by both accumulation studies with known megalin ligand FITC-albumin and viability assays using the nephrotoxic aminoglycoside antibiotic gentamicin, also known megalin ligand (Marzolo and Farfán 2011; Moestrup et al. 1995). The introduced in vitro model was used to study megalin-mediated cell accumulation of a 15-mer anti-VEGFR peptide (15-mer) labeled with either ^68^Ga or ^99m^Tc.

## Methods

### Cell lines

Human kidney epithelial cell line HK2 (CRL-2190, ATCC) naturally expressing *LRP2* was maintained in Dulbecco’s Modified Eagle’s Medium supplemented with 10% fetal bovine serum (FBS). The human glioblastoma cancer U-87 MG cell line (89081402, ECACC) was cultured in DMEM supplemented with 10% FBS, 1% non-essential amino acids (NAA), 2 mM L-glutamine, and 1 mM sodium pyruvate. The human ovarian cancer SK-OV-3 cell line (HTB-77, ATCC) was maintained in McCoy's 5A medium supplemented with 10% FBS, 1% NAA, and 1 mM sodium pyruvate. The media and all supplements were purchased from Merck. The cells were incubated in a humidified atmosphere containing 5% CO_2_ at 37 °C.

### Designed sgRNAs

**Fig. 1:**
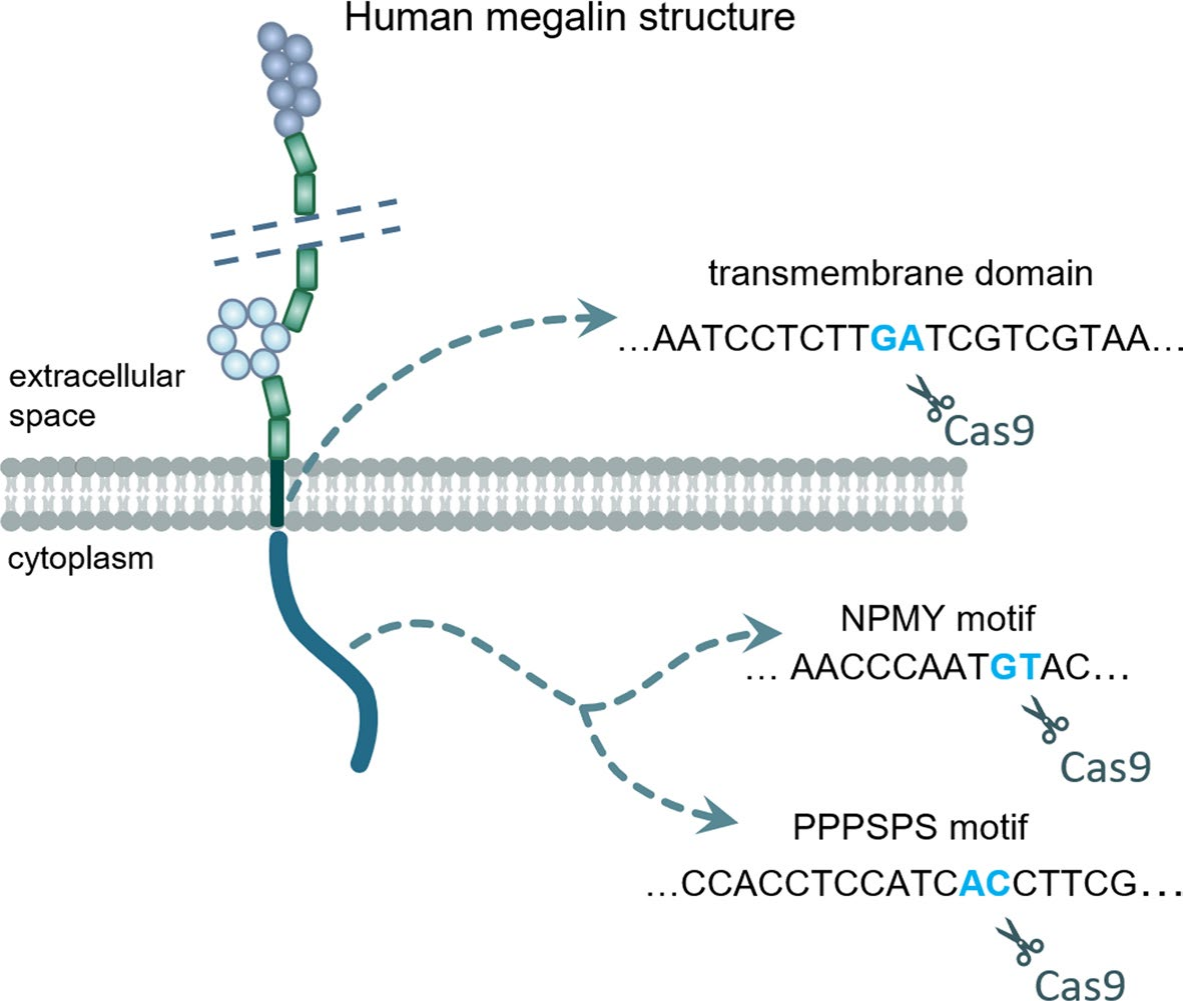
Visualization of human megalin structure and designed cuts. Selected sgRNA sequences targeting the transmembrane domain (TMD), motifs important in regulation of function and ligands trafficking (NPMY motif), and basal phosphorylation of the receptor (PPPSPS motif)

The single guide RNAs (sgRNAs; Synthego Corporation, Table 1) were designed using an online designer tool by the Genetic Perturbation Platform (Broad Institute) by submitting three sequences of the *LRP2* gene: the transmembrane domain (TMD), asparagine-proline-methionine-tyrosine (NPMY) motif, and proline-proline-proline-serine-proline-serine (PPPSPS) motif (Fig. 1). See the Additional file 1 for details.

**Table 1:** Designed sgRNA sequences

Target	Designed sequence
TMD	CTCCA ATTAC GACGA TCAAG
NPMY motif	CTGTC TCTGG CTGAG TACAT
PPPSPS motif	TTAGC AGGGA GCGAA GGTGA

### CRISPR/Cas9 knockout of *LRP2*

**Fig. 2:**
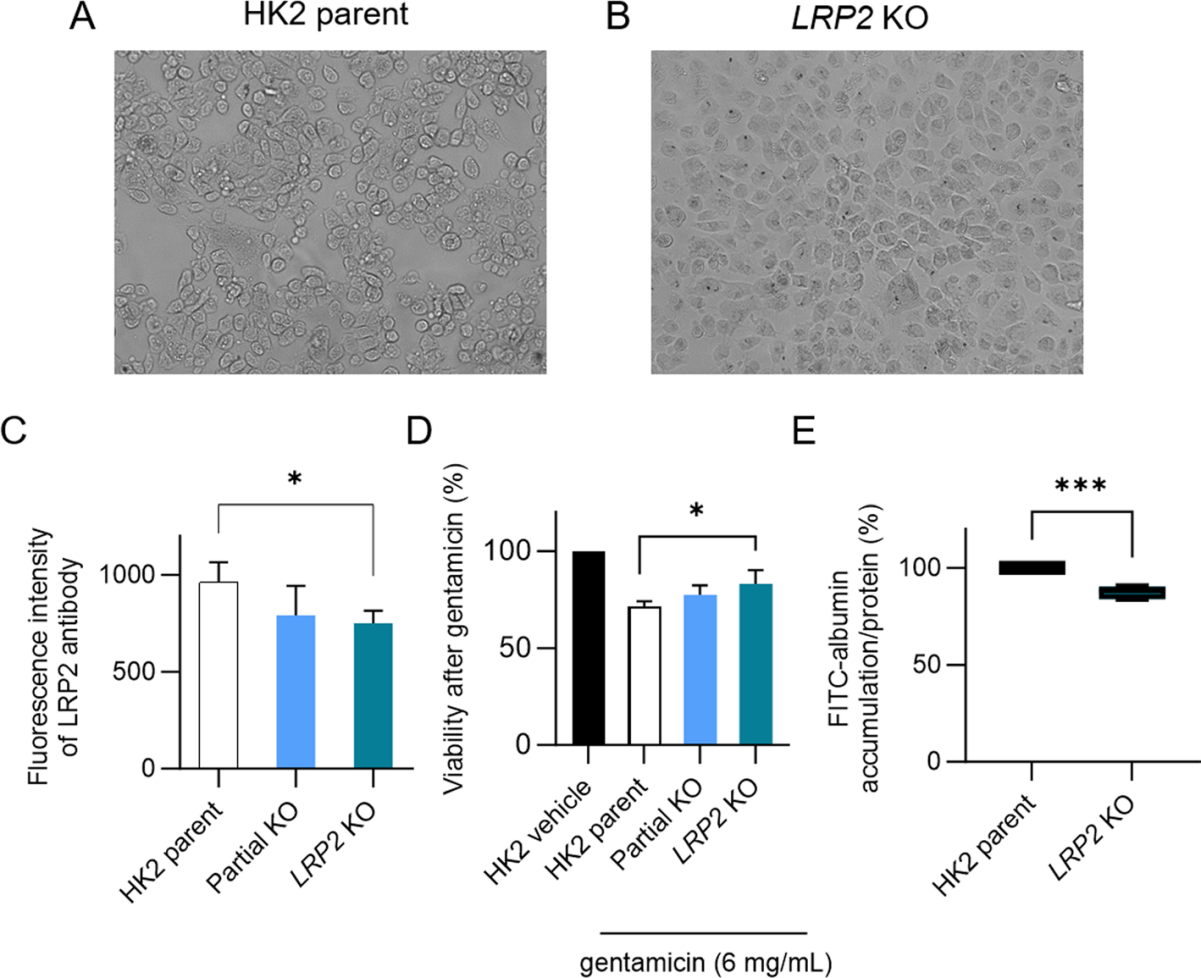
Verification of *LRP2* edits by flow cytometry analysis, gentamicin and FITC-albumin accumulation. **A** Morphological changes in parent HK2 and **B** *LRP2* KO cells observed using the FLoid™ cell imaging station after treatment with cytotoxic doses of gentamicin (48 h, 6 mg/mL). **C** Cells were incubated with 0.05 mg/mL megalin polyclonal antibody for 2 h followed by CF™ 594 secondary antibody staining (1h, 5 µg/mL). The fluorescence intensity was quantified using a median based on fluorescent histograms of the cell populations. **D** Viability of edited and parent HK2 cells was evaluated after 48 h treatment with cytotoxic doses of gentamicin (6 mg/mL), with the graph representing the relative cellular viability. **E** FITC-albumin accumulation studies (24 µg/mL) were performed after 2 h incubation. One-way ANOVA with Dunnett’s post hoc test or *t-*test were implemented to compare differences in parent and edited HK2 cells, *P < 0.05, ***P ≤ 0.001

The CRISPR/cas9 gene editing technique was implemented to generate *LRP2* KO HK2 cells (Fig. 1) employing designed sgRNA sequences (Table 1). HK2 cells were seeded on 24-well plate (10^5^ cells/well). Cells were transfected by sgRNA (720 ng per reaction) and TrueCut™ Cas9 Protein v2 (3 840 ng per reaction; Invitrogen) using Lipofectamine™ CRISPRMAX™ Cas9 Transfection Reagent (Invitrogen) according to the manufacturer’s instructions 24 h after seeding. The knockout reactions were repeated twice for each sgRNA. Each transfection was performed after 48 h of recovery. The final group of cells transfected sequentially six times was designated as *LRP2* KO. A group of cells transfected only once with NPMY sgRNA but twice with TMD and PPPSPS sgRNAs, was designated as partial KO (Figs. 2A–E, 5A and B).

### Viability assay

The cells were seeded in a 96-well plate (10^4^ cells per well) 24 h prior to the experiment. The cells treated with gentamicin at cytotoxic concentration (6 mg/mL) were incubated in parallel with the controls in standard culture medium (representing 100% cell viability). The cells cultivated with 10% DMSO in culture medium represent 0% cell viability, which was measured after 48 h using the CellTiter 96® AQueous One Solution Cell Proliferation Assay (Promega) according to the manufacturer’s protocol. Absorbance was detected using a spectrophotometric plate reader (TECAN Infinite M200).

### Accumulation studies

The accumulation studies of fluorescein isothiocyanate-labelled albumin (FITC-albumin, Merck) were performed in 24-well plates (10^5^ cells per well). The cells were incubated with 24 µg/mL FITC-albumin, and Passive Lysis Buffer (Promega) was added to lyse the cells after 2 h treatment. The accumulated FITC-albumin was quantified by measuring the fluorescence intensity of cell lysates (Tecan Spark® multimode microplate reader). Fluorescence intensity was plotted against total protein concentration determined by the commercially available Pierce™ BCA Protein Assay Kit (ThermoFisher Scientific).

The accumulation studies of 15-mers were performed in 24-well plates (10^5^ cells per well). The cells were treated with either 20 µg/mL ^68^Ga-radiolabeled 15-mer or 27 µg/mL ^99m^Tc-radiolabeled 15-mer for 2 h. The accumulated radioactivity was determined using an automatic γ counter (2480 Wizard2, PerkinElmer), with results presented as counts per minute related to the amount of cell protein in each sample.

### Flow cytometry analysis of megalin expression

The harvested cells (parent HK2 and *LRP2* KO cells, 10^6^ cells) were treated with bovine serum albumin (Merck) blocking buffer (5% in PBS) for 90 min. The blocked cells were incubated with 0.05 ng/mL megalin polyclonal antibody (bs-3909R, Bioss Antibodies) for 2 h followed by secondary anti-rabbit CF™ 594antibody staining (5 µg/mL, 1h; SAB4600099-250UL, Merck). The cell viability was determined by Hoechst 33342 (ThermoFisher Scientific) staining for 30 min. The megalin antibody binding was analyzed by flow cytometry using the SA3800 spectral analyzer (Sony Biotechnology). Expression was determined based on medians of the fluorescence histograms for the parent and *LRP2* edited cells.

### Fluorescence-activated cell sorting (FACS)

The cells were seeded in a 6-well plate (4 × 10^5^ cells/well) 48 h before FACS sorting, then treated with FITC-albumin (megalin ligand, 24 µg/mL, 2 h) and rhodamine B (viability dye, 48 µg/mL, 15 min). The cells exerting affected megalin function, rhodamine B-positive/FITC-albumin-negative (Additional file 1: Fig. 1B), were sorted by MA900 Multi-Application Cell Sorter (Sony Biotechnology) using the Sony Sorting Chip-130µm for MA900 (Sony Biotechnology). The sorted cells were designated as *LRP2* KO1 and *LRP2* KO2 based on the stringency of sorting conditions (threshold set at 5% and 2% of the lowest FITC-albumin fluorescence intensity, respectively). The cells were collected in a cultivating medium enriched by 30% FBS and 1% Penicillin–Streptomycin (Merck).

### Laser scanning confocal microscopy

The cells were seeded in 4-well cell imaging slides (4 × 10^5^ cells/well). After 48 h incubation cells were fixed using a solution of 4% paraformaldehyde in PBS (15 min) at room temperature (RT). Cells were treated with BSA blocking buffer (5% in PBS) for 90 min at RT, then the cells were incubated on ice with Megalin polyclonal antibody (0,01 mg/mL, 4 h) followed by incubation with secondary anti-rabbit CF™ 594 antibody (5 µg/mL, 1 h). In the next step, the cells were stained with Hoechst 33342 (1 µg/mL, 30 min, ThermoFisher Scientific) to visualize nuclei. To stain the actin filaments ActinGreen 488 ReadyProbes reagent (ThermoFisher Scientific) was used according to the manufacturer’s instructions. Excess salts were removed by washing with demineralized water (5 min). Dry samples were mounted with ProLong Gold antifade reagent (Invitrogen), dried, nail-polished and analyzed by Nikon A1 + confocal system (Nikon) equipped with NIS Elements AR 4.20 software (Laboratory Imaging).

### Western blotting

The harvested cells (parent HK2, LRP2 KO cells, U-87 MG, and SC-OV-3) were lysed in RIPA lysis buffer (Merck) containing protease and phosphatase inhibitors cocktail (Roche). Protein concentration was measured using the Pierce™ BCA Protein Assay Kit (ThermoFisher Scientific), samples were diluted in 4 × Laemmli sample buffer (BioRad) supplemented with mercaptoethanol (Merck) and heated at 95 °C for 10 min. Equal amounts of protein were separated on 4–20% Mini-PROTEAN® TGX Stain-Free™ Protein Gels (10 well, BioRad), transferred onto TransBlot Turbo Midi-size PVDF Membrane (BioRad), and blocked with EveryBlot Blocking Buffer (10 min, BioRad). The membrane was incubated with primary LRP2 Polyclonal Antibody (1:500, 1 h, 19700–1-AP, ThermoFisher) followed by the incubation with Goat anti-Rabbit IgG (H + L) Secondary Antibody, HRP (1:200 000, 1 h, 31460, ThermoFisher). The other membrane was incubated with beta Actin Recombinant Rabbit Monoclonal Antibody (JF53-10), HRP (1:10 000, 1 h, MA5-32540, ThermoFisher). Chemiluminescence signals were detected using SuperSignal™ West Femto Maximum Sensitivity Substrate (ThermoFisher) and visualized by the ChemiDoc MP Imaging system (BioRad).

### Computational biomolecular docking of 15-mers with megalin

The crystal structures of megalin (PDB ID: 8EM4) were obtained from the protein data bank database (PDB) (Beenken et al. 2023). The high ambiguity-driven protein–protein docking (HADDOCK) v2.4 platform was used for computational biomolecular interaction studies (protein-peptide). The generated 15-mer/megalin complex was further visualized and analyzed by the PyMOL Molecular Graphics System (Schrödinger) (Zundert et al. 2016).

Active residues for the receptors were selected based on the CASTp v3.0 server and flexible peptide-protein docking with HPEPDOCK 2.0 (Tian et al. 2018; Zhou et al. 2018). The amino acid residues R828, W844, F845, R846, W870, N872, F889, H914, W930, R931, Y1206, D1209, and T1248 (binding sites of megalin) were used as active residues in the peptide-protein docking with 15-mer.

The obtained HADDOCK results of the 15-mer/megalin complexes were used to predict the values of the binding affinity (ΔG) (kcal·mol^−1^) and dissociation constant [nM] at 37.0 °C using the online server tool PROtein binDIng enerGY prediction (PRODIGY) (Xue et al. 2016).

### Synthesis of peptides

The peptide consisting of 15 amino acids (15-mer) was custom synthesized (Apigenex) according to a previously published structure (Basile et al. 2011). The peptide was then conjugated with 2-[1,4,7-Triazacyclononan-1-yl-4,7-bis(tBu-ester)]-1,5-pentanedioic acid (NODAGA) for ^68^ Ga-labeling or was modified on the N-terminus with lysine (K), aspartic acid (D), and cysteine (C) for ^99m^Tc-labeling. Detailed protocol is provided in the Additional file 1.

### Peptide radiolabeling

^68^ Ga-labeling of 15-mer was performed in strict metal-free conditions according to a previously published protocol (Knetsch et al. 2011). The final activity of the products was 0.74 MBq·nmol^−1^. ^99m^Tc-labeling of 15-mer was performed according to a previously published protocol (Cazzamalli et al. 2016). The final activity of the products was 0.14 MBq·nmol^−1^ (Fig. 5A). Detailed protocol is provided in the Additional file 1.

### In Vitro stability

Both radiolabeled peptides were tested for stability in Opti-Minimal Essential Medium (Opti-MEM; Gibco) at 37 °C at the time intervals 0, 30, 60, 90 and 120 min after radiolabeling.

^68^Ga-labeled 15-mer and ^99m^Tc-labeled 15-mer were separately added into medium in the ratio 1:4, and incubated at 37 °C. At the above specified time points, stability aliquots (100 µL) were analyzed using high-performance liquid chromatography with radiometric detection of radiochemical purity.

### Statistical analysis

The data were statistically analyzed using GraphPad Prism 9.5.1 (GraphPad Software), with the results presented as the mean ± SD from three independent measurements performed in biological triplicates. A paired *t*-test or ANOVA with Dunnett’s post hoc test was used to compare the data in the tested edited cells to intact HK2 control cells. The P value < 0.05 was considered statistically significant.

## Results

### Flow cytometry confirms decreased megalin level in CRISPR/Cas9-modified cells

To monitor the effect of CRISPR/Cas9 edits, the parent HK2 cells and CRISPR/Cas9-modified cells (*LRP2 KO*) (Fig. 2A and B) labeled with the antibody against megalin were analyzed using flow cytometry. The histograms of fluorescence intensity showed a shift in peak area in the CRISPR/Cas9-modified cells compared to the parent HK2 cells representing a change in megalin expression (Additional file 1: Fig. 1A). Thus, the analysis revealed decreased expression of megalin in the KO cells (Fig. 2C).

### The reduction of sensitivity to gentamicin cytotoxic effect in KO cells

A viability assay was applied to indirectly confirm the CRISPR/Cas9 knockout effect on megalin function with the megalin ligand gentamicin, used in cytotoxic concentration. The modified cells (partial KO or *LRP2* KO) exerted higher viability after treatment compared to HK2 parent cells. Higher survival due to lower internalization of the cytotoxic gentamicin indicates suppressed megalin function. The viability of modified cells increased with an increasing number of transfections and knockout targets (Fig. 2D). The loss of cellular membrane asymmetry, apoptotic bodies and washed-out dead cells were observed by the FLoid™ cell imaging instrument in intact HK2 cells (Fig. 2A), whereas the edited HK2 cells showed no morphological changes (Fig. 2B).

### Reduced FITC-albumin accumulation by KO cells

FITC-albumin, another megalin ligand, was employed in accumulation studies to verify the functioning of the CRISPR/Cas9 knockout. Reduced FITC-albumin accumulation in KO cells confirmed the validity of our model for further testing of potential megalin ligands (Fig. 2E). FITC-albumin accumulation was also used in subsequent FACS sorting of KO cells (Additional file 1: Fig. 1B). Sorted KO cell subpopulations exerted decreased FITC-albumin accumulation (Additional file 1: Fig. 1C).

### Reduced megalin protein level in KO cells

**Fig. 3:**
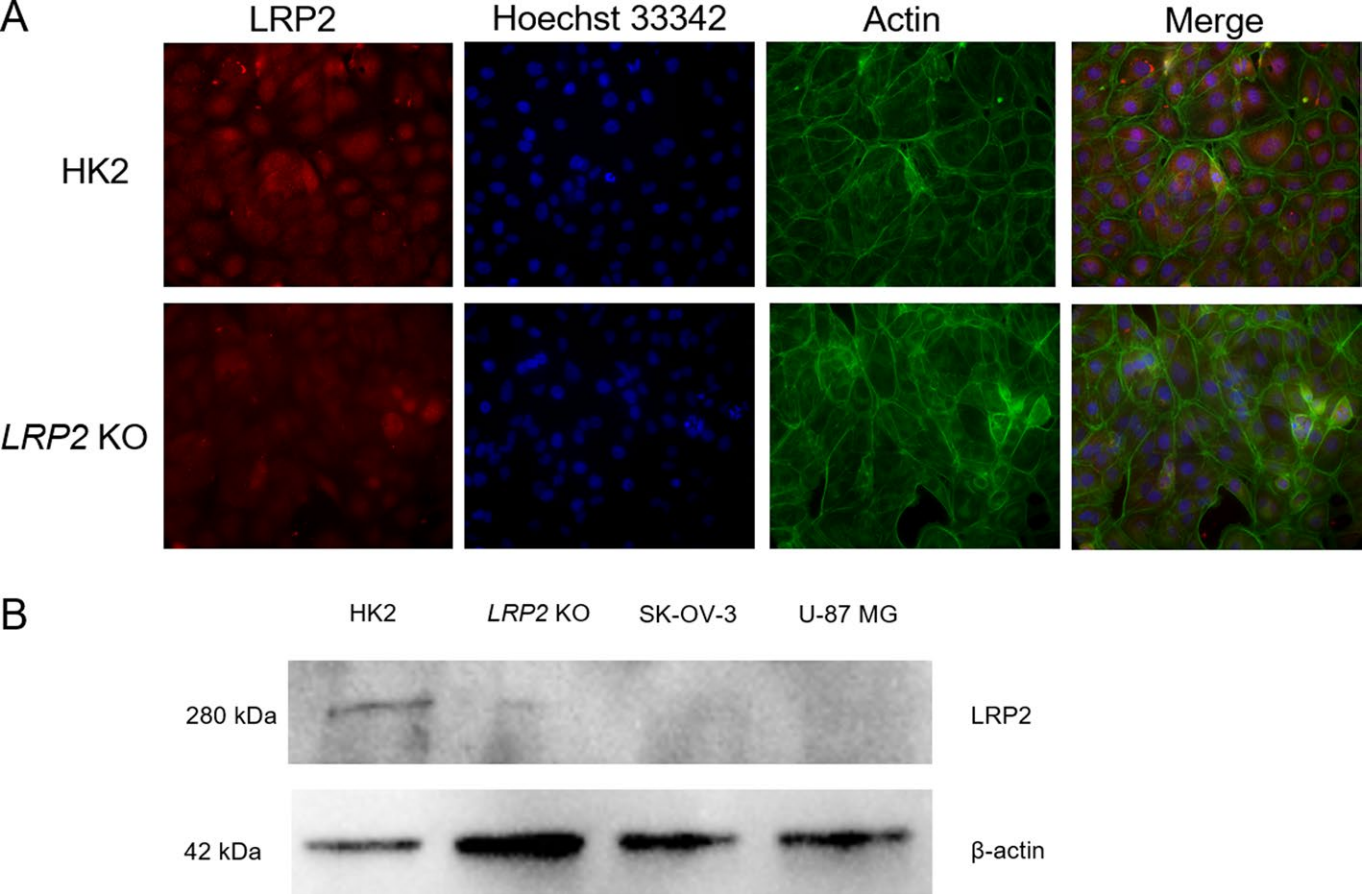
Verification of LRP2 knockout by western blotting analysis and laser scanning confocal microscopy. **A** HK2 cells expressing *LRP2* and *LRP2* KO cells shown by confocal microscopy. Cells were incubated with megalin polyclonal antibody (red), Hoechst 33342 to visualize the nuclei of viable cells (blue) and ActinGreen™ 488 ReadyProbes was used for cytoskeleton imaging (green). **B** Western blot analysis of LRP2 knockout using the parent HK2, *LRP2* KO cells, and negative controls without *LRP2* expression, SK-OV-3 and U-87 MG cells

To compare the megalin protein expression of modified and parent HK2 cells, western blotting and laser scanning confocal microscopy were employed. The decreased megalin antibody binding was detected using confocal microscopy (Fig. 3A) suggesting decreased megalin expression. The western blotting confirmed reduced protein level of megalin in modified *LRP2* KO cells in comparison with parent HK2 cells normalized to β-actin (Fig. 3B, Additional file 1: Fig. 2), SK-OV-3 and U-87MG cells were used as negative controls.

### Biomolecular docking determines 15-mers as megalin ligands

**Fig. 4:**
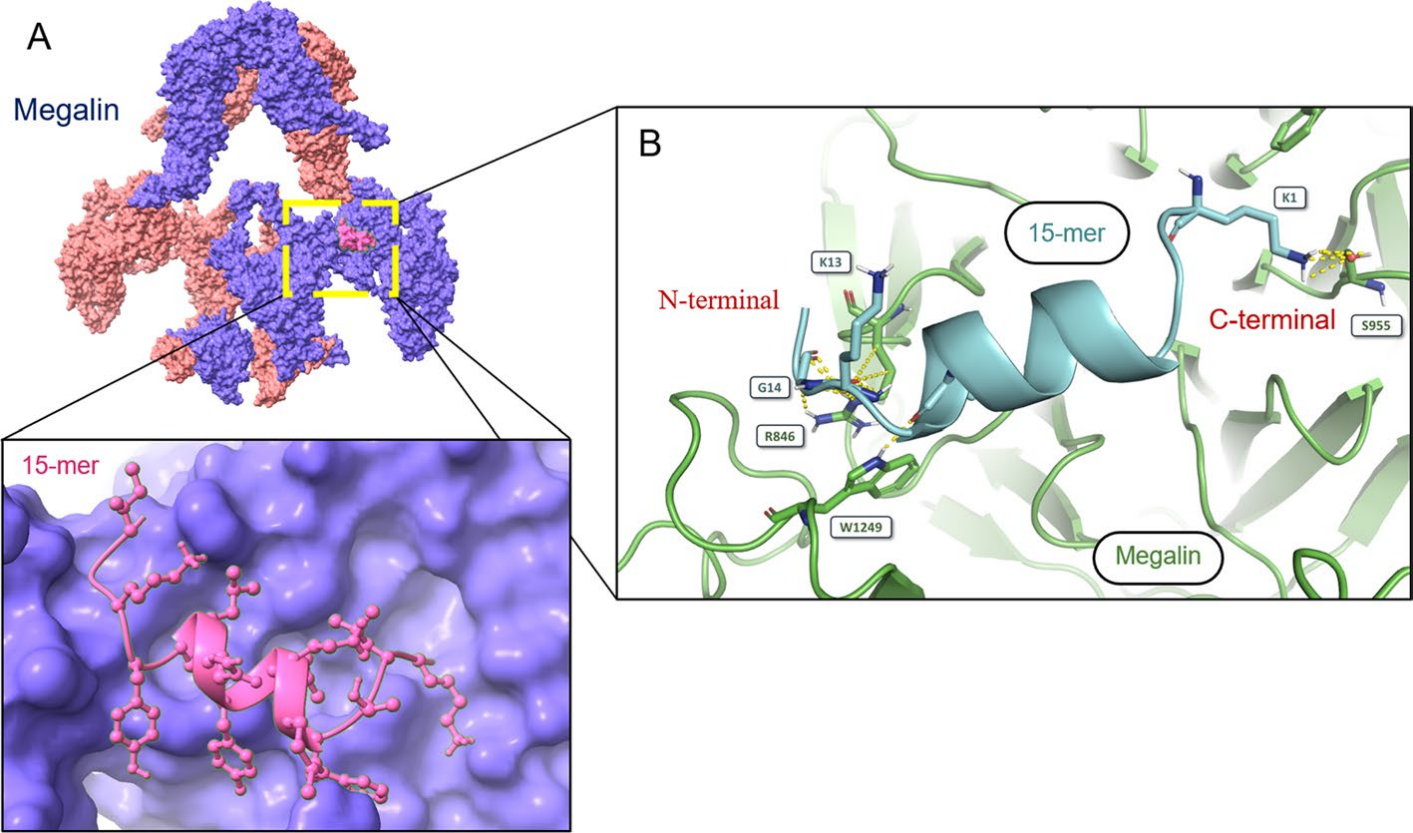
Molecular visualization of the 15-mer/megalin complex based on crystallographic data. **A** The complex of 15-mer (pink) and megalin. **B** The 3D model of 15-mer (turquoise) and megalin interaction with highlighted hydrogen bonds (yellow) and distinct residues

Protein-peptide docking simulations were used to investigate the interaction between the 15-mer and megalin (Fig. 4), guided by experimental constraints (Beenken et al. 2023; Zundert et al. 2016). The potential active sites of megalin were determined by flexible peptide-protein docking and geometric and topological analysis (Tian et al. 2018; Zhou et al. 2018). 15-mer docked to megalin with high affinity (HADDOCK docking score: − 114.3 ± 1.0; (Table 2)). The interaction was mediated by hydrophobic and hydrogen bond contacts driven by van der Waals forces (− 52.3 ± 2.6 kcal·mol^−1^) and electrostatic interactions (− 88.0 ± 22.7 kcal·mol^−1^). The 15-mer bound to the active site of megalin with high affinity (K_d_ = 220 nM; (Table 2)). These values are consistent with the in vitro assays, demonstrating interactions between 15-mer and megalin.

**Table 2:** Docking scores, energy values, and binding affinities of unlabeled 15-mer toward megalin

HADDOCK (High Ambiguity Driven protein–protein DOCKing)						HPEPDOCK		PRODIGY Binding affinity	
Receptor	Peptide	HADDOCK score	Van der Waals energy (kcal·mol^−1^)	Electrostatic energy (kcal·mol^−1^)	Desolvation energy (kcal·mol^−1^)	Z -Score	Docking energy score	∆G (kcal·mol^−1^)	K_d_ (M) at 37.0 °C
Megalin	15-mer	− 114.3 ± 1.0	− 52.3 ± 2.6	− 88.0 ± 22.7	− 53.6 ± 1.5	− 1.6	− 282.95	− 9.4	2.2·10^–7^

### In vitro stability testing of [^68^Ga]Ga-15-mer and [^99m^Tc]Tc-15-mer

**Fig. 5:**
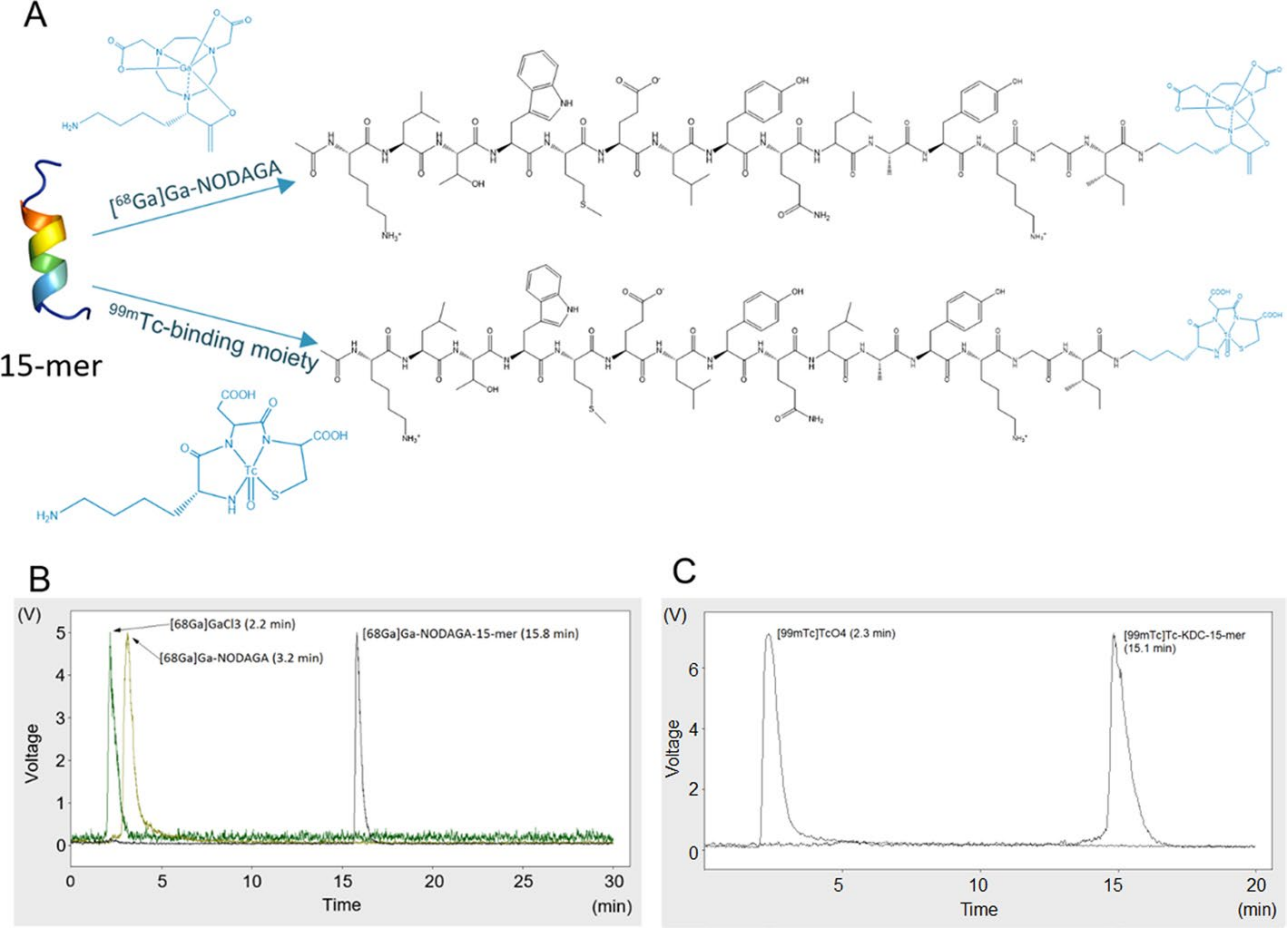
Radiolabeling of 15-mer and purity evaluation. **A** 15-mer radiolabeled by two different approaches, [^68^Ga]Ga-NODAGA or ^99m^Tc-binding moiety (KDC). The example radiochromatogram overlays of ^68^Ga containing compounds (**B**) and ^99m^Tc containing compounds (**C**) with the indicated retention times

The radiolabeling of both peptides resulted in 100% radiochemical purity. The prepared radiopeptides were further tested for stability in an Opti-MEM medium. The chromatographic records of the peptides shortly after radiolabeling showed the retention times of the peaks for [^68^Ga]Ga-NODAGA-15-mer at 15.8 min (Fig. 5B) and [^99m^Tc]Tc-KDC-15-mer at 15.1 min (Fig. 5C). There was no presence of radiochemical impurities such as free ^68^Ga (t_R_ = 2.2 min) or [^68^Ga]Ga-NODAGA (t_R_ = 3.2 min) in the ^68^Ga-labelled preparation, and no free ^99m^Tc (t_R_ = 2.3 min) in ^99m^Tc-labelled preparation.

Results of radiolabeled 15-mers stability found in the Opti-MEM are summarized in Table 3. [^99m^Tc]Tc-KDC-15mer remained stable for more than 1 h. The found impurity corresponded to free ^99m^Tc, probably in hydrolyzed form (t_R_ = 2.3 min). [^68^Ga]Ga-NODAGA-15-mer revealed lower stability compared to [^99m^Tc]Tc-KDC-15mer. Degradation in the Opti-MEM resulted in the release of free ^68^Ga, which was detected at t_R_ = 2.2 min. No other peaks besides those of the radiopeptides and free forms of radiometals were detected in radiochromatograms (Additional file 1: Figs 3 and 4).

**Table 3:** Stability testing of [^68^Ga]Ga-NODAGA-15-mer and [^99m^Tc]Tc-KDC-15-mer in Opti-MEM

Incubation time [min]	[^68^Ga]Ga-NODAGA-15-mer [%]	[^99m^Tc]Tc-KDC-15-mer [%]
0	100	100
20	100	100
40	100	100
60	100	100
80	44.2	100
100	57.8	49.6
120	45.4	55.4

### Megalin plays a role in radiolabeled 15-mers internalization

**Fig. 6:**
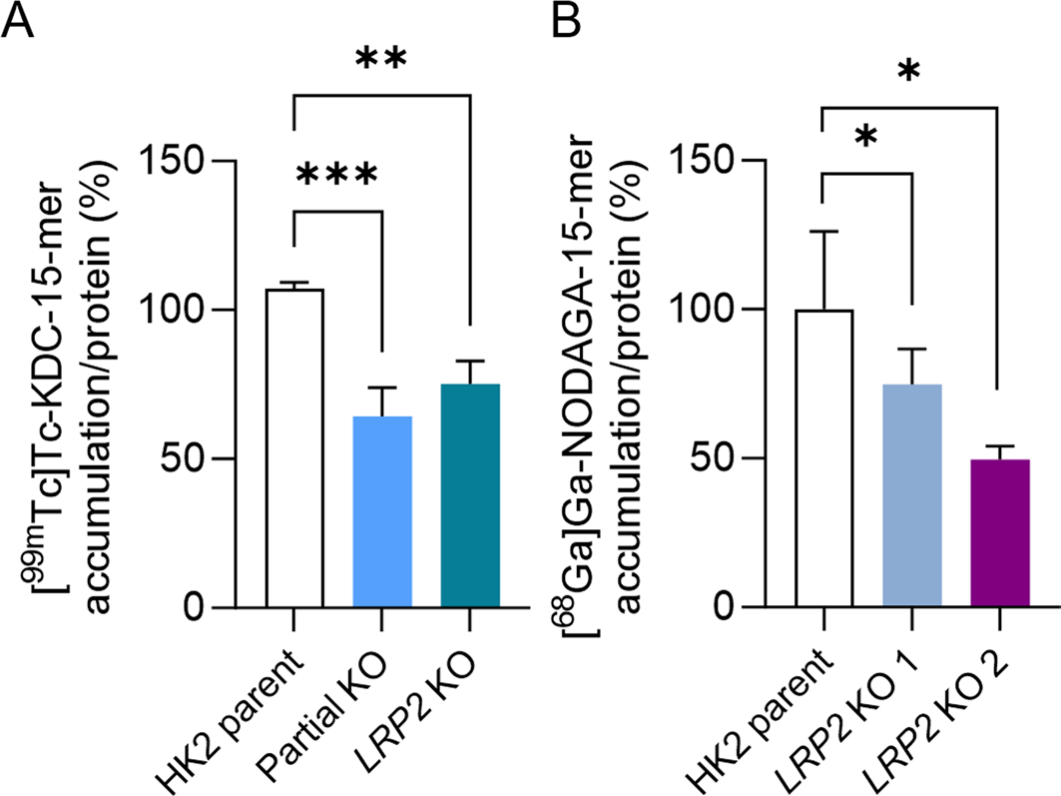
Accumulation of radiolabeled 15-mer. The [^68^Ga]Ga-NODAGA-15-mer and [^99m^Tc]Tc-KDC-15-mer internalization assays in HK2 parent, partial KO and *LRP2* KO cells. The accumulation of radiolabeled 15-mers measured in parent HK2 cells was set as 100%. All the data were normalized to the total protein level. **A** The HK2 parent, partial KO and *LRP2* KO cells were treated with [^99m^Tc]Tc-KDC-15-mer (2 h, 27 µg/mL, 37 °C). The experimental data were obtained from three independent experiments performed in biological triplicates. **B** The [^68^Ga]Ga-NODAGA-15-mer (2 h, 20 µg/mL, 37 °C) treatment was performed in HK2 parent, *LRP2* KO 1 and *LRP2* KO 2 cells in biological triplicates to examine ^68^Ga-radiolabeled 15-mer megalin-mediated accumulation. ANOVA with Dunnett’s post hoc test was used to compare KO models with control cells, *P ≤ 0.05, **P ≤ 0.01

15-mer was labeled using either ^99m^Tc-binding moiety (KDC) or [^68^Ga]-chelating NODAGA (Fig. 5A). Decreased accumulation of both ^99m^Tc-labeled (Fig. 6A) and ^68^Ga-labeled (Fig. 6B) 15-mer was detected in both partial KO and *LRP2* KO cells. Thus, the radioligand labeling did not show a significant effect on 15-mer internalization by megalin in the cellular model. This effect corresponds with biomolecular docking results (Table 2) showing 15-mer as the megalin ligand and confirming megalin role in the internalization of these radiolabeled 15-mers.

## Discussion

The application of radiolabeled receptor-specific peptides can be associated with nephrotoxicity induced by the accumulation of radionuclides in renal cortex (Geenen et al. 2021). Clinical application of ^177^Lu- or ^90^Y-labeled radiopharmaceuticals has resulted in significant renal radiotoxicity in patients treated with targeted radiotherapy (Otte et al. 1999; Kwekkeboom et al. 2008). The nephrotoxic effect can limit clinically applicable doses and, consequently, the effectiveness of the treatment (Geenen et al. 2021). Moreover, the ongoing introduction of alpha emitters with stronger radiotoxic effects as radionuclides in targeted radiotherapy will place even more importance on assessments of the potential nephrotoxicity of candidate radiopharmaceuticals.

Although renal cortex accumulation is the underlying mechanism of renal radiotoxicity described in radiolabeled peptides or other radiolabeled compounds (Jong et al. 2004), the molecular mechanisms have been uncovered recently. Several studies have shown the accumulation of radiolabeled peptides via the megalin receptor (encoded by the *LRP2* gene). Ex vivo and in vivo experiments in rats have indicated the involvement of megalin transport in the renal accumulation of [^111^In]In-DTPA-octreotide, [^177^Lu]Lu-DOTA-octreotate, and [^111^In]In-DOTA-octreotate (Melis et al. 2005). Biodistribution studies of several ^111^In-labeled peptides using a KO murine in vivo model showed lower radioactivity levels in megalin-deficient kidneys than in megalin-expressing kidneys (Vegt et al. 2011; Jong et al. 2005; Melis et al. 2010). Although studies on megalin involvement in the radiopeptide nephrotoxicity in vivo aimed at other radiolabeled peptides are not available, it can be expected that the mechanism of nephrotoxicity in analogical radiopeptides may be similar to that of ^111^In-labeled compounds. Nevertheless, the experiments using in vivo KO models can confirm radiopeptides as megalin ligands only indirectly by describing changes in renal radiopeptide biodistribution. Moreover, animal testing is not only limited due to ethical and economic reasons, but also interspecies diversity must be considered.

Drug transport studies in vitro generally employ comparisons of two cell groups: one with low/knockout expression of the relevant gene, and another with high/induced expression of the evaluated transporter. Such systems can detect the relationship between transporter expression and transport of the compounds tested. However, an analogical approach for megalin ligands determination in vitro is not available for a human kidney model due to megalin complex structure. Therefore, the *LRP2* KO model seems to be a valuable tool for future studies assessing radiolabeled compounds as possible megalin ligands.

The introduced CRISPR/Cas9 editing represents a convenient approach to generate a knockout cellular model based on a human kidney cell line with high endogenous megalin expression. Since the HK2 cell line possesses all the necessary attributes, including the human kidney origin, high expression of *LRP2* and easy handling, HK2 was selected for genetic modifications (Ryan et al. 1994). The sgRNAs were specifically designed to target key structures in the *LRP2* gene (Fig. 1) to knockout its function and thus affect ligand internalization (Marzolo and Farfán 2011). In a recent study, a homozygous *LRP2* knockout human embryonic stem cell line was generated using the CRISPR/Cas9 technique, although the authors do not show its application in any megalin functional study (You et al. 2021). In addition, *Lrp2*-knockout OK cells have been produced using the CRISPR/Cas9 technique (Long et al. 2022; Rbaibi et al. 2023). While OK cells are proximal tubular epithelial cells, this cell line is isolated from the Virginia opossum, thus does not represent a human cellular model.

The introduction of our model was verified by accumulation of two known megalin ligands, FITC-albumin and gentamicin (Moestrup et al. 1995; Cui et al. 1996). The accumulation of both ligands was decreased in the KO cells compared to the parent HK2 cell line confirming the *LRP2* gene editing. Nevertheless, we observed an increase in FITC-albumin accumulation with a longer cultivation time of KO cells. This phenomenon could be explained by the inhibition of nutrient uptake into the KO cells via megalin (Marzolo and Farfán 2011) and/or incomplete transfection of cells in the culture. Thus, the parent cells may exhibit higher viability, resulting in their overgrowth in the cell culture, while KO cells may proliferate slower. To solve this problem, cell sorting was employed to separate KO cells from parent cells without the need for further gene modifications. Moreover, the megalin expression changes were confirmed on the protein level by western blotting and by confocal microscopy depicting lower fluorescence of KO cells compared to the intact HK2.

The validated method has already been introduced in radiolabeled peptide accumulation studies. The 15-mer peptide was selected due to the fact its molecular weight corresponds to the size of proved megalin ligands (Vegt et al. 2011; Basile et al. 2011). The 15-mer derived from vascular endothelial growth factor A (VEGF-A) N-terminal helix showed potent antiangiogenic effects in both in vitro and in vivo studies (Basile et al. 2011). 15-mer was labeled either via NODAGA, a bifunctional chelating component, or via KDC, a ^99m^Tc-binding moiety, to assess possible structure modification effect on megalin binding.

To simulate kidney reabsorption in vitro, the HK2, immortalized proximal tubular cells, and their *LRP2* KO counterparts were used in internalization experiments. The decreased accumulation found in the KO cells confirmed both tested radiopeptides as ligands of megalin endocytic receptor irrespective of the method used for radiolabeling. This finding is in accordance with the relatively broad ligand spectrum of the megalin, which includes proteins with various structures and molecular weight (Goto et al. 2023; Melis et al. 2010). The megalin mediates endocytosis in renal proximal tubules of different substances such as albumin, insulin, apolipoproteins, enzymes, hormones, and aminoglycoside antibiotics (Marzolo and Farfán 2011; Goto et al. 2023). Considering the data on peptide or protein transport via the megalin receptor, an analogically high number of radiopeptides with heterogenic structures can be expected to be potential megalin ligands. Therefore, the introduced in vitro method for the determination of radiolabeled peptide interaction with megalin may become an important tool in future preclinical testing of radiopharmaceuticals. The radiolabeled peptides identification as megalin ligands may contribute to optimization during candidate selection with the goal of limiting the risk of renal radiopeptide accumulation and radiotoxicity.

## Conclusion

*LRP2* knockout HK2 model was introduced as a potential experimental tool for head-to-head preclinical testing of radiolabeled compound accumulation. Moreover, it can serve as a valuable tool in nephrotoxic mechanism clarification. Employing the *LRP2* knockout model, both candidate anti-VEGFR radiopeptides were identified as megalin ligands.

### Supplementary Information

**Additional file 1**. The additional file contains information pertaining to the detailed methodology of sgRNA design, peptide synthesis, radiolabeling and purity control, additional results of flow cytometry analysis and FACS sorting, full-length Western blot images, and radiochromatograms of both peptides.

## Data Availability

The datasets used and analyzed during the current study are available from the corresponding author on reasonable request.

## Abbreviations

BCA: Bicinchoninic acid assay
CRISPR/Cas9: Clustered regularly interspaced palindromic repeats/CRISPR-associated protein 9
DMSO: Dimethyl sulfoxide
FACS: Fluorescence-activated cell sorting
FBS: Fetal bovine serum
FITC-albumin: Fluorescein isothiocyanate-labelled albumin
HADDOCK: High ambiguity-driven protein–protein docking
KO: Knockout
LRP2: Low-density lipoprotein receptor-related protein 2
NAA: Non-essential amino acids
NODAGA: 2-[1,4,7-Triazacyclononan-1-yl-4,7-bis(tBu-ester)]-1,5-pentanedioic acid
PRODIGY: PROtein binDIng enerGY
sgRNA: Single guide RNA
TMD: Transmembrane domain
VEGF(R): Vascular endothelial growth factor (receptor)

## References

[CR1] Akizawa H, Uehara T, Arano Y (2008). Renal uptake and metabolism of radiopharmaceuticals derived from peptides and proteins. Adv Drug Deliv Rev.

[CR2] Basile A, Del Gatto A, Diana D, Di Stasi R, Falco A, Festa M (2011). Characterization of a designed vascular endothelial growth factor receptor antagonist helical peptide with antiangiogenic activity in vivo. J Med Chem.

[CR3] Beenken A, Cerutti G, Brasch J, Guo Y, Sheng Z, Erdjument-Bromage H (2023). Structures of LRP2 reveal a molecular machine for endocytosis. Cell.

[CR4] Cazzamalli S, Dal Corso A, Neri D (2016). Acetazolamide serves as selective delivery vehicle for dipeptide-linked drugs to renal cell carcinoma. Mol Cancer Ther.

[CR5] Cui S, Verroust PJ, Moestrup SK, Christensen EI (1996). Megalin/gp330 mediates uptake of albumin in renal proximal tubule. Am J Physiol.

[CR6] De Jong M, Valkema R, Van Gameren A, Van Boven H, Bex A, Van De Weyer EP (2004). Inhomogeneous localization of radioactivity in the human kidney after injection of [(111)In-DTPA]octreotide. J Nucl Med.

[CR7] de Jong M, Barone R, Krenning E, Bernard B, Melis M, Visser T (2005). Megalin is essential for renal proximal tubule reabsorption of (111)In-DTPA-octreotide. J Nucl Med.

[CR8] de Roode KE, Joosten L, Behe M (2024). Towards the magic radioactive bullet: improving targeted radionuclide therapy by reducing the renal retention of radioligands. Pharmaceuticals (basel).

[CR9] Geenen L, Nonnekens J, Konijnenberg M, Baatout S, De Jong M, Aerts A (2021). Overcoming nephrotoxicity in peptide receptor radionuclide therapy using [(177)Lu]Lu-DOTA-TATE for the treatment of neuroendocrine tumours. Nucl Med Biol.

[CR10] Goto S, Hosojima M, Kabasawa H, Saito A (2023). The endocytosis receptor megalin: from bench to bedside. Int J Biochem Cell Biol.

[CR11] Knetsch PA, Petrik M, Griessinger CM, Rangger C, Fani M, Kesenheimer C (2011). [68Ga]NODAGA-RGD for imaging αvβ3 integrin expression. Eur J Nucl Med Mol Imaging.

[CR12] Kręcisz P, Czarnecka K, Królicki L, Mikiciuk-Olasik E, Szymański P (2021). Radiolabeled peptides and antibodies in medicine. Bioconjug Chem.

[CR13] Kwekkeboom DJ, de Herder WW, Kam BL, van Eijck CH, van Essen M, Kooij PP (2008). Treatment with the radiolabeled somatostatin analog [177 Lu-DOTA 0, Tyr3]octreotate: toxicity, efficacy, and survival. J Clin Oncol.

[CR14] Long KR, Rbaibi Y, Bondi CD, Ford BR, Poholek AC, Boyd-Shiwarski CR (2022). Cubilin-, megalin-, and Dab2-dependent transcription revealed by CRISPR/Cas9 knockout in kidney proximal tubule cells. Am J Physiol Renal Physiol.

[CR15] Marzolo MP, Farfán P (2011). New insights into the roles of megalin/LRP2 and the regulation of its functional expression. Biol Res.

[CR16] Melis M, Krenning EP, Bernard BF, Barone R, Visser TJ, de Jong M (2005). Localisation and mechanism of renal retention of radiolabelled somatostatin analogues. Eur J Nucl Med Mol Imaging.

[CR17] Melis M, Vegt E, Konijnenberg MW, de Visser M, Bijster M, Vermeij M (2010). Nephrotoxicity in mice after repeated imaging using 111In-labeled peptides. J Nucl Med.

[CR18] Moestrup SK, Cui S, Vorum H, Bregengård C, Bjørn SE, Norris K (1995). Evidence that epithelial glycoprotein 330/megalin mediates uptake of polybasic drugs. J Clin Invest.

[CR19] Otte A, Herrmann R, Heppeler A, Behe M, Jermann E, Powell P (1999). Yttrium-90 DOTATOC: first clinical results. Eur J Nucl Med.

[CR20] Rbaibi Y, Long KR, Shipman KE, Ren Q, Baty CJ, Kashlan OB, Weisz OA (2023). Megalin, cubilin, and Dab2 drive endocytic flux in kidney proximal tubule cells. Mol Biol Cell.

[CR21] Ryan MJ, Johnson G, Kirk J, Fuerstenberg SM, Zager RA, Torok-Storb B (1994). HK-2: an immortalized proximal tubule epithelial cell line from normal adult human kidney. Kidney Int.

[CR22] Tian W, Chen C, Lei X, Zhao J, Liang J (2018). CASTp 3.0: computed atlas of surface topography of proteins. Nucleic Acids Res.

[CR23] van Zundert GCP, Rodrigues J, Trellet M, Schmitz C, Kastritis PL, Karaca E (2016). The HADDOCK2.2 web server: user-friendly integrative modeling of biomolecular complexes. J Mol Biol.

[CR24] Vegt E, Melis M, Eek A, de Visser M, Brom M, Oyen WJ (2011). Renal uptake of different radiolabelled peptides is mediated by megalin: SPECT and biodistribution studies in megalin-deficient mice. Eur J Nucl Med Mol Imaging.

[CR25] Volková M, Mandíková J, Bárta P, Navrátilová L, Lázníčková A, Trejtnar F (2015). The in vivo disposition and in vitro transmembrane transport of two model radiometabolites of DOTA-conjugated receptor-specific peptides labelled with (177) Lu. J Labelled Comp Radiopharm.

[CR26] Xue LC, Rodrigues JP, Kastritis PL, Bonvin AM, Vangone A (2016). PRODIGY: a web server for predicting the binding affinity of protein-protein complexes. Bioinformatics.

[CR27] You J, Cheng Y, Yang XJ, Chen L (2021). Generation of a homozygous LRP2 knockout human embryonic stem cell line (FDCHDPe010-A-56) by CRISPR/Cas9 system. Stem Cell Res.

[CR28] Zhou P, Jin B, Li H, Huang SY (2018). HPEPDOCK: a web server for blind peptide-protein docking based on a hierarchical algorithm. Nucleic Acids Res.

