# Supplementary material for: Radiolabeled 15-mer peptide internalization is mediated by megalin (LRP2 receptor) in a CRISPR/Cas9-based LRP2 knockout human kidney cell model
Source: EJNMMI Radiopharm Chem. 2024 Apr 18;9:32. doi: 10.1186/s41181-024-00262-2 (PMC11026318; doi:10.1186/s41181-024-00262-2)
Supplement: Supplementary file 1 — Additional file 1. The additional file contains information pertaining to the detailed methodology of sgRNA design, peptide synthesis, radiolabeling and purity control, additional results of flow cytometry analysis and FACS sorting, full-length Western blot images, and radiochromatograms of both peptides. [file 41181_2024_262_MOESM1_ESM.docx]

**Radiolabeled 15-mer peptide internalization is mediated by megalin (LRP2 receptor) in a CRISPR/Cas9-based *LRP2* knockout human kidney cell model**

Anna Durinova^1^, Lucie Smutna^1*^, Pavel Barta^2^, Rajamanikkam Kamaraj^1^, Tomas Smutny^1^, Bernhard Schmierer^3^, Petr Pavek^1^, Frantisek Trejtnar^1^

*^1^Department of Pharmacology and Toxicology, Faculty of Pharmacy in Hradec Kralove, Charles University, Hradec Kralove, Czech Republic; ^2^Department of Biophysics and Physical Chemistry, Faculty of Pharmacy in Hradec Kralove, Charles University, Hradec Kralove, Czech Republic; ^3^CRISPR Functional Genomics, SciLifeLab and Department of Medical Biochemistry and Biophysics, Karolinska Institutet, Solna, Sweden*

** Corresponding author*

**Supplementary Information**

**METHODS**

**Designed sgRNAs**

The single guide RNAs (sgRNAs) were designed (Table 1) using the online designer tool by the Genetic Perturbation Platform (Broad Institute) by submitting three sequences of *LRP2* gene: transmembrane domain (TMD), asparagine-proline-methionine-tyrosine (NPMY) motif (the DNA sequence was prolonged by 27 bases before and after the motif sequence) and proline-proline-proline-serine-proline-serine (PPPSPS) motif (the DNA sequence was prolonged by 24 bases before and after the motif sequence). The selection of specific guide sequences was realized considering a high on-target efficacy score, low possible off-target effect and position of the cut. Designated sgRNAs were synthesized by the Synthego Corporation.

**Synthesis of Peptides**

The peptide consisting of 15 amino acids was custom synthesized according to a previously published (*13*) structure (acetyl-KLTWMELYQLAYKGI-amide) and conjugated with bifunctional chelator 2-[1,4,7-Triazacyclononan-1-yl-4,7-bis(tBu-ester)]-1,5-pentanedioic acid (NODAGA) (Macrocyclics) to prepare the final NODAGA-15-mer peptide (APIGENEX). The prepared NODAGA-15-mer was analyzed by UPLC MS (Waters Acquity TUV Detector,) with the found product chemical purity over 95 %.

The 18 amino acid peptide was custom synthesized (APIGENEX) according to a previously published (*13*) structure (acetyl-KLTWMELYQLAYKGI-amide) with a small modification on N-terminus on which ^99m^Tc-binding moiety consisting of three more amino acids (lysine, aspartic acid, and cysteine) was added. The final product acetyl-KLTWMELYQLAYKGIKDC-OH was analyzed by UPLC MS (Waters Acquity TUV Detector) with the found product chemical purity over 96 %.

**^68^Ga-Labeling and Radiochemical Purity Control**

^68^Ga-labeling of 15-mer was performed in strict metal‑free conditions according to a modified previously published protocol (*14*). Briefly, an sodium acetate solution (1.9 M, 30 µL) and [^68^Ga]Ga^3+^Cl of the main fraction of the germanium-68/gallium-68 generator (Eckert & Ziegler Radiopharma GmbH) eluate (5 MBq in 0.1 M HCl, approx. 300 µL) were added to NODAGA-15-mer (1 μg/μL). The final activity of the products was 0.74 MBq·nmol^-1^. The mixture was incubated for 15 min at room temperature. Thanks to the radiolabeling method optimization, there was no need for [^68^Ga]Ga-NODAGA-15-mer purification.

Radiochemical purity was analyzed using high-performance liquid chromatography (HPLC system Agilent 1100 Series, Agilent Technologies Inc). Distilled water (0.1 % TFA) and acetonitrile (0.1 % TFA) as components of the mobile phase (flow rate 1.0  mL·min^-1^, gradient elution 0 to 80 % respectively in 30-min run) and the ZORBAX Eclipse XDB‑C18 4.6 × 150 mm (Agilent Technologies Inc.) column were used for the separation with a radiometric detection. The analysis of the prepared peptide proceeded after their radiolabeling (referred to as 0 h).

**^99m^Tc-Labeling and Radiochemical Purity Control**

The ^99m^Tc**-**radiolabeling of 15-mer was performed according to a modified previously published protocol (*15*). Briefly, binding moiety-15-mer (5.8 nmol) in PBS (50 µL, pH 7.4) was added into solution (150 µL) of sodium glucoheptonate (20 mg, Merck) with SnCl_2_ (300 µg, Merck). Tris-buffered saline (600 µL, pH 6.69) was added and the final mixture was degassed for 5 min with nitrogen gas. The addition of ^99m^Tc-eluate (0.8 MBq, 1.2 µL, GE Healthcare) binding moiety-15-mer mixture followed and the radiolabeling reaction proceeded for 20 min at 90 °C. When ^99m^Tc-labeling was over, the mixture was cooled down. The prepared radioligand [^99m^Tc]Tc-15-mer was analyzed on radiochemical purity using high-performance liquid chromatography (HPLC system Agilent 1100 Series, Agilent Technologies Inc.). Distilled water (0.1 % TFA) and acetonitrile (0.1 % TFA) as components of the mobile phase (flow rate 1.0 mL·min^-1^, gradient elution 0 to 80 % respectively in 20-min run) and the ZORBAX Eclipse XDB C18 4.6 × 150 mm (Agilent Technologies Inc.) column were used for the separation with a radiometric detection. The analysis of ^99m^Tc-labeled 15-mer proceeded after radiolabeling (referred to as 0 h).

**RESULTS**

**Flow cytometry analysis and FACS sorting**


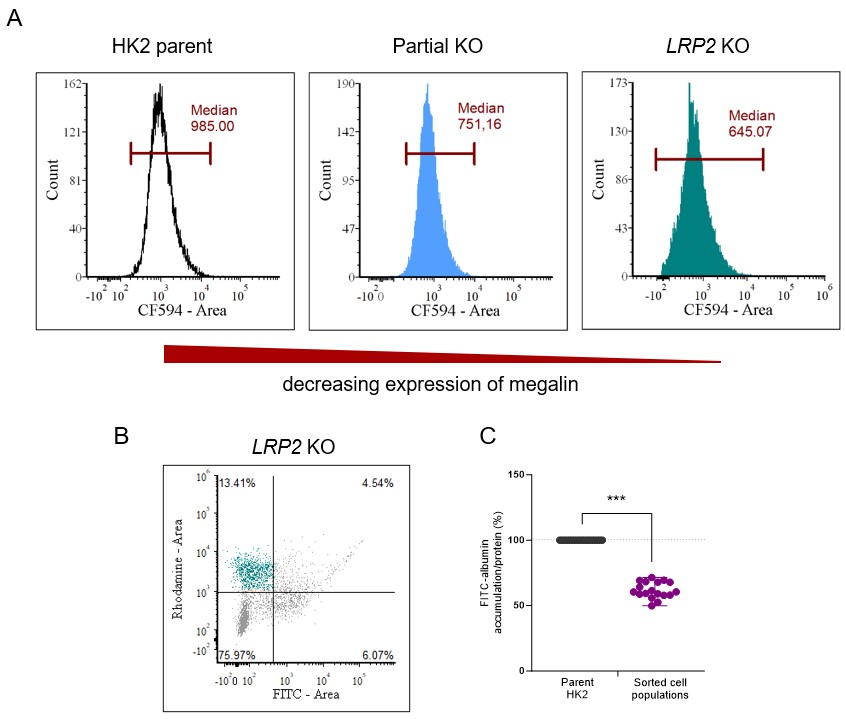


**Supplemental Figure 1**. **Flow cytometry analysis and FACS sorting.** (A) Cells were incubated with Megalin polyclonal antibody (0.05 mg/mL, 2 h) followed by incubation with CF™ 594 secondary antibody (1h, 5 µg/mL). Representative histograms of median fluorescence intensity were obtained from HK2 parent, partial KO, and *LRP2* KO cells. (B) Cells were treated with FITC-albumin (megalin ligand, 24 µg/mL, 2 h) and rhodamine B (viability dye, 48 µg/mL, 15 min). FACS sorted rhodamine B-positive/FITC-albumin-negative cells are highlighted in the upper left quadrant of the scatter plot. (C) FITC-albumin accumulation studies (24 µg/mL, 2 h) were performed in sorted cell populations.

**Western blot analysis**


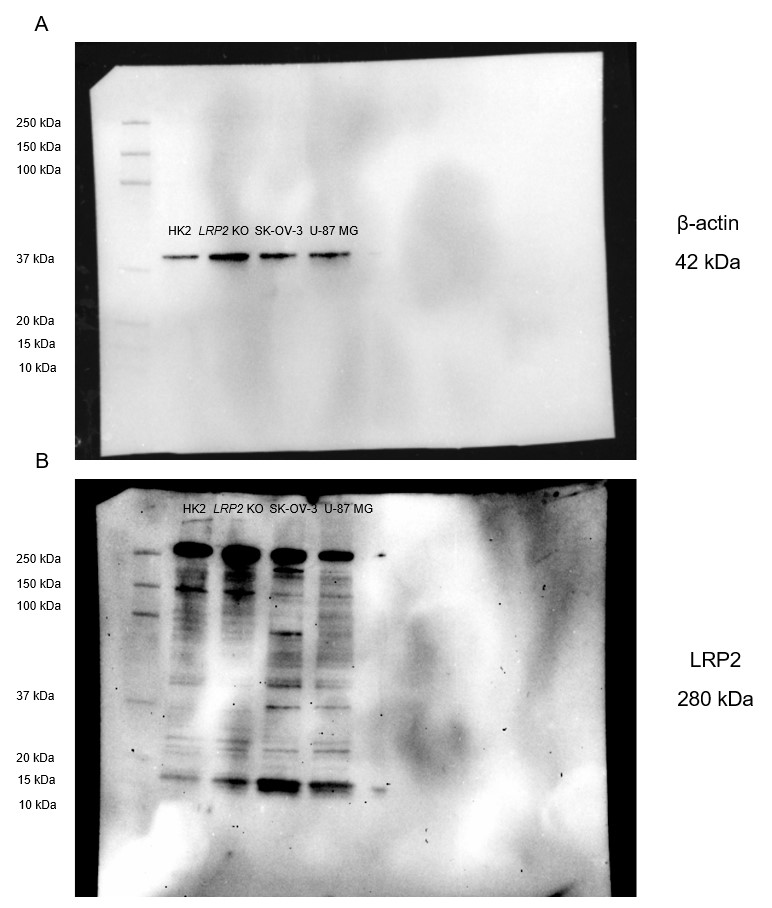


**Supplemental Figure 2**. **Western blot analysis.** (A) Full length blot for loading control β-actin (exposition time 14 s). (B) Full length blot for LRP2. HK2 cells exerting a high level of LRP2, modified *LRP2* KO cells and negative controls without LRP2 expression, SK-OV-3 and U-87 MG cells were employed in western blot (exposition time 500 s). Specific gels were used for the analysis of each protein.

**The stability of [^68^Ga]Ga-NODAGA-15-mer**

A

B

C

D

E

F

G

H


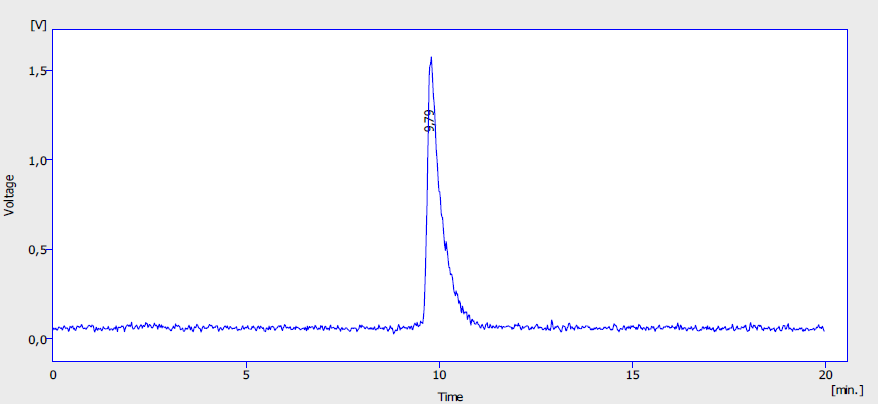

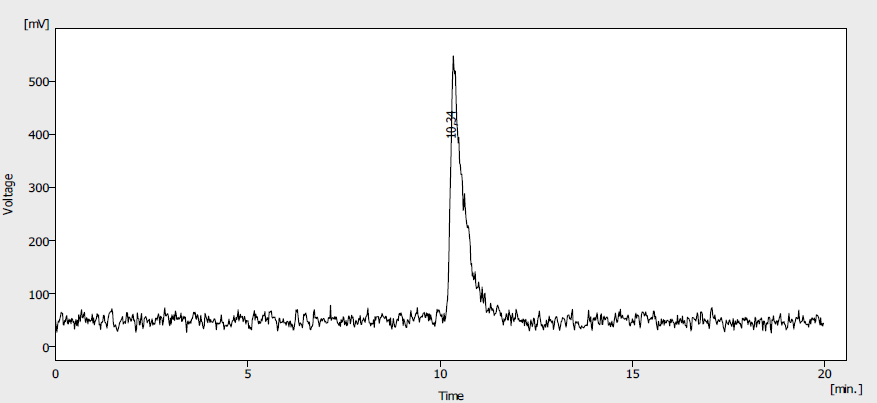

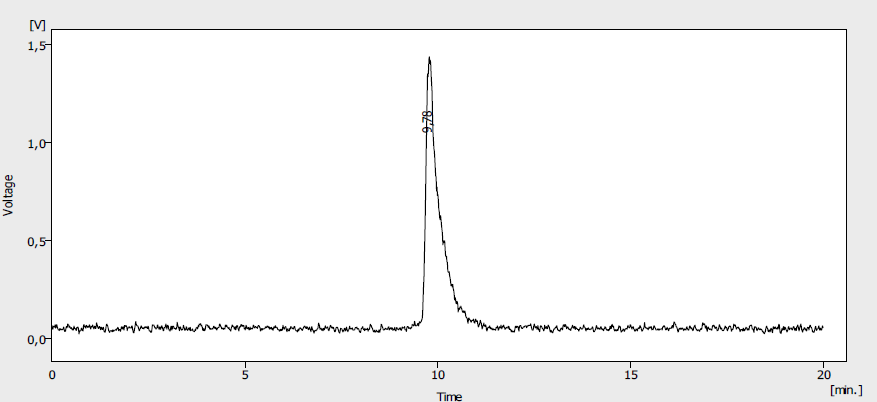

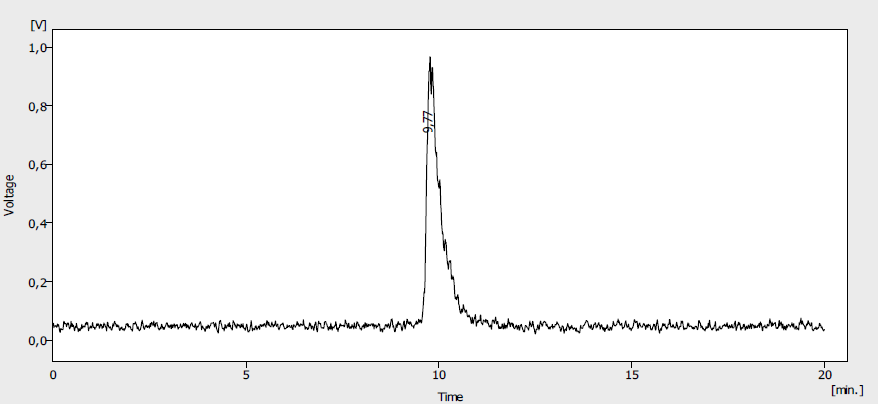

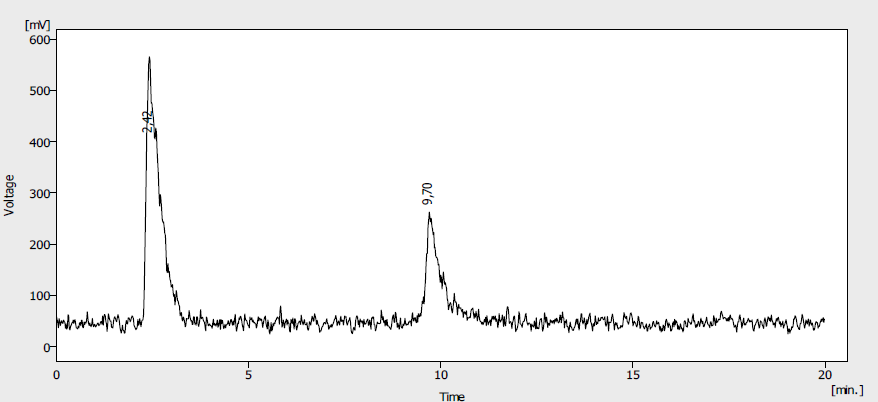

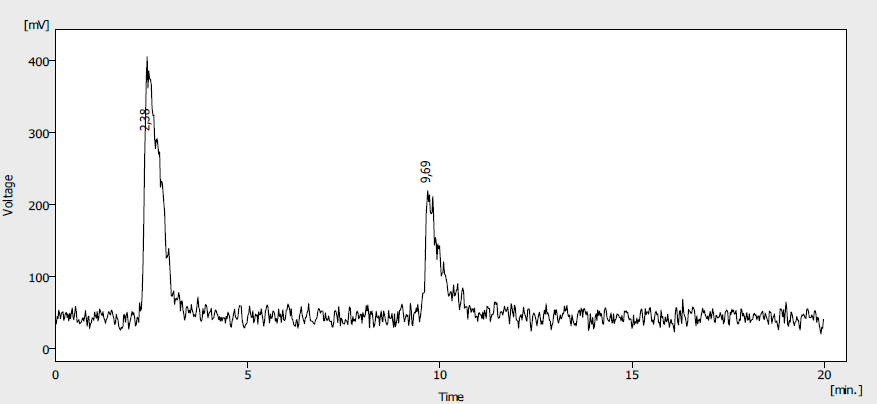

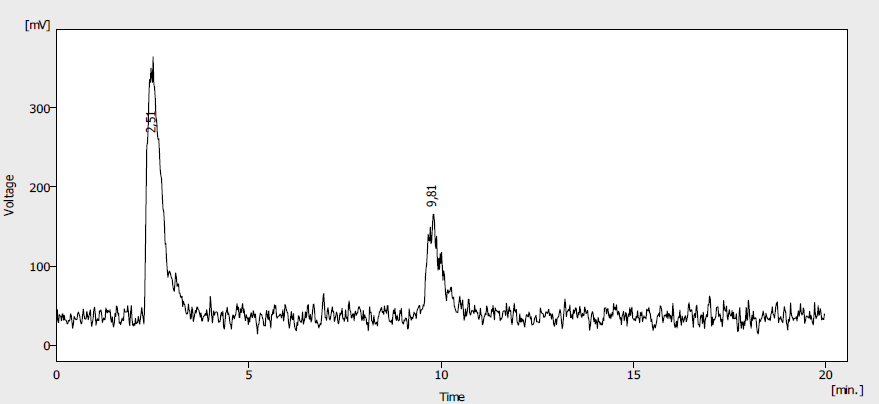

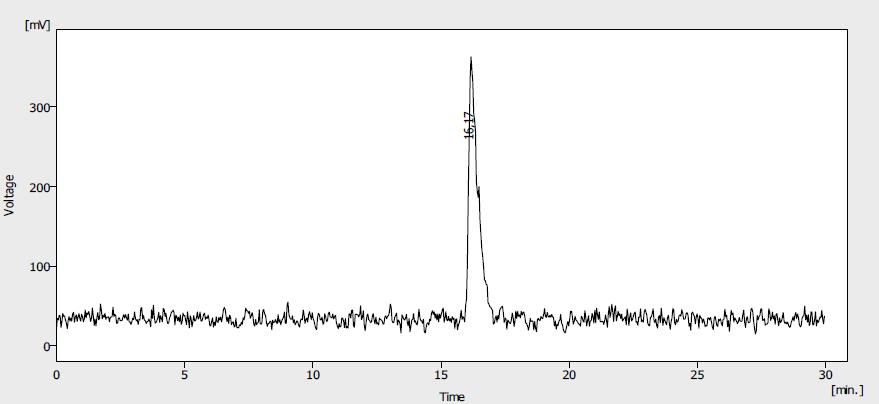


**Supplemental Figure 3. Radiochromatograms of** **[^68^Ga]Ga-NODAGA-15-mer**. Radiochromatograms of ^68^Ga-labeled 15-mer gained right after the radiolabeling (A) 0 min, or after incubation in Opti-MEM (B) 20 min, (C) 40 min, (D) 60 min, (E) 80 min, (F) 100 min, and (G) 120 min. The (H) represents radiochromatogram of [^68^Ga]Ga-NODAGA-15-mer after 120 min incubation in phosphate-buffered saline (pH 7.4). The run time was set to 20 min (A-G) resp. 30 min (H).

**The stability of [^99m^Tc]Tc-KDC-15-mer**


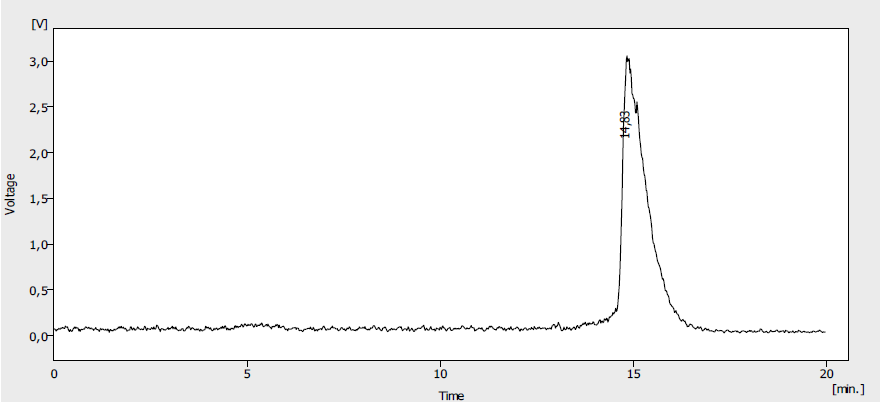


A


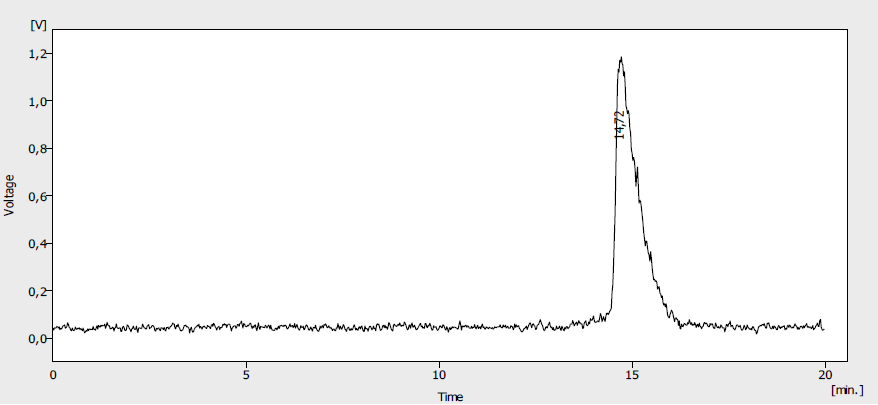


B

C

D

E

F

G

H


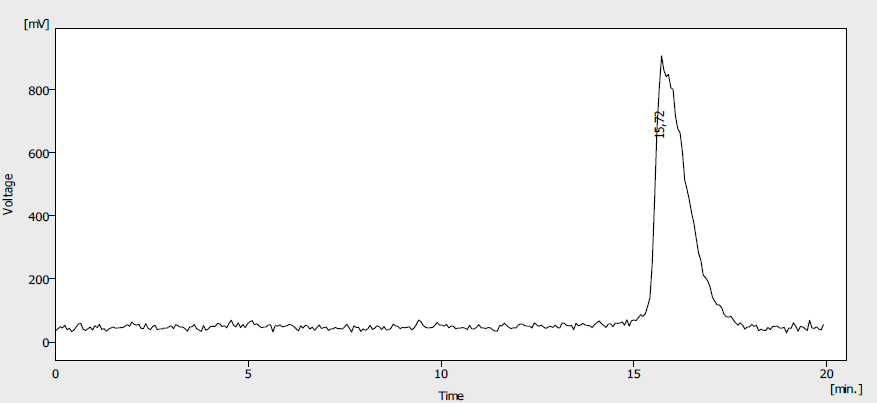

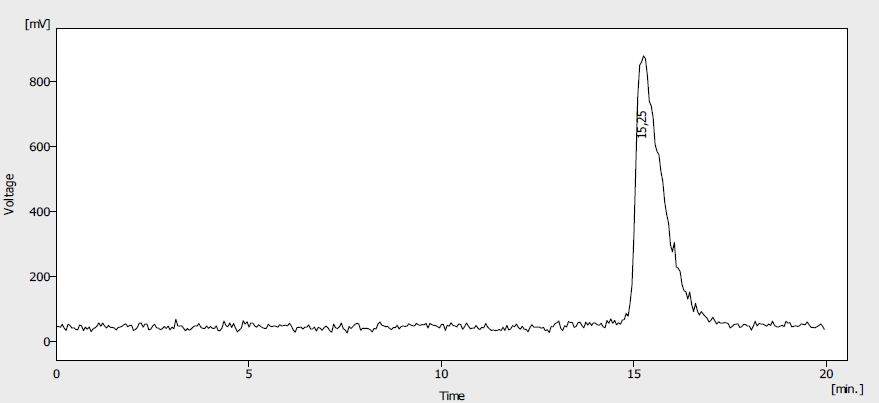

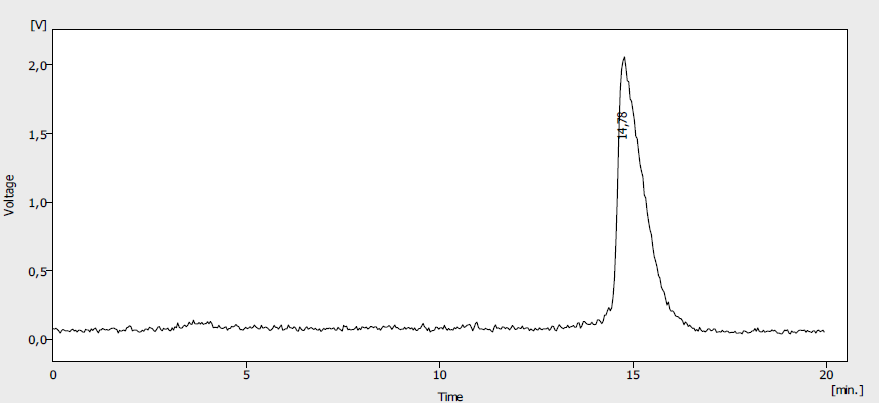

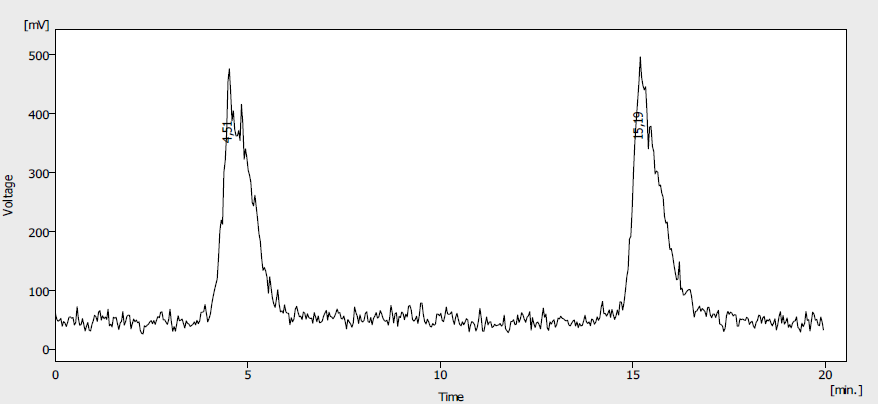

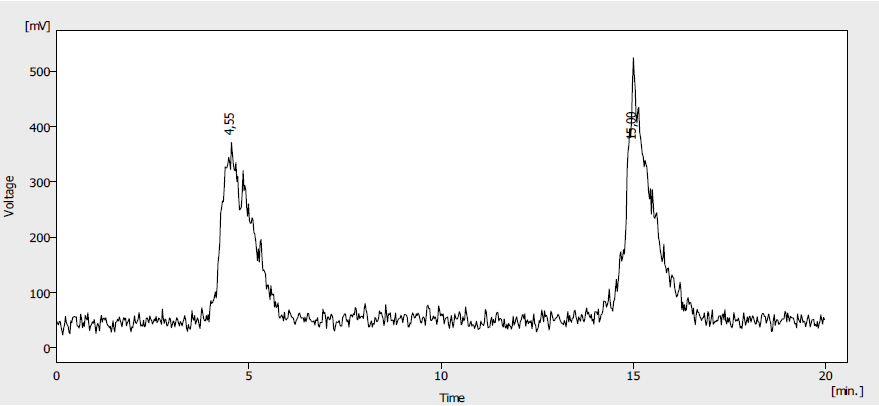

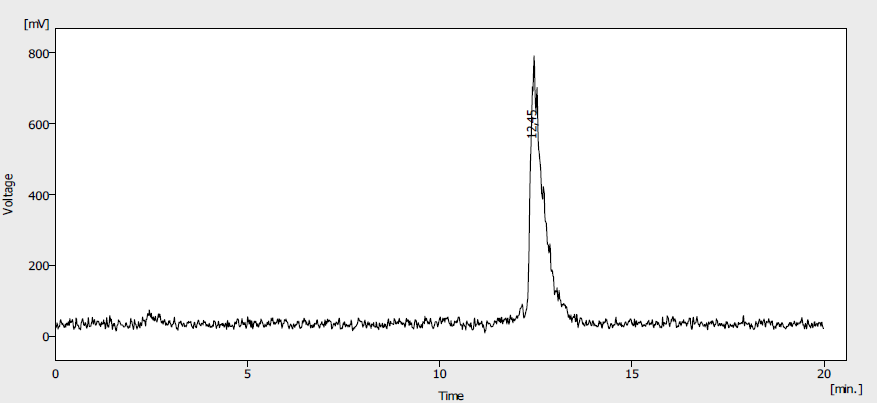


**Supplemental Figure 4. Radiochromatograms of [^99m^Tc]Tc-KDC-15-mer.** Radiochromatograms of ^99m^Tc-labeled 15-mer gained right after the radiolabeling (A) 0 min, or after incubation in Opti-MEM (B) 20 min, (C) 40 min, (D) 60 min, (E) 80 min, (F) 100 min, and (G) 120 min. The (H) represents radiochromatogram of [^99m^Tc]Tc-KDC-15-mer after 5 hours incubation in phosphate-buffered saline (pH 7.4). The run time was set to 20 min.
